# ShadowY: a dark yellow fluorescent protein for FLIM-based FRET measurement

**DOI:** 10.1038/s41598-017-07002-4

**Published:** 2017-07-28

**Authors:** Hideji Murakoshi, Akihiro C. E. Shibata

**Affiliations:** 1 Supportive Center for Brain Research, National Institute for Physiological Sciences, Okazaki, Aichi 444-8585 Japan; 2 Department of Physiological Sciences, Sokendai (The Graduate University for Advanced Studies), Okazaki, Aichi 444-8585 Japan; 3 Precursory Research for Embryonic Science and Technology, Japan Science and Technology Agency (JST), Kawaguchi, Saitama 332-0012 Japan

## Abstract

Fluorescence lifetime imaging microscopy (FLIM)-based Förster resonance energy transfer (FRET) measurement (FLIM-FRET) is one of the powerful methods for imaging of intracellular protein activities such as protein–protein interactions and conformational changes. Here, using saturation mutagenesis, we developed a dark yellow fluorescent protein named ShadowY that can serve as an acceptor for FLIM-FRET. ShadowY is spectrally similar to the previously reported dark YFP but has a much smaller quantum yield, greater extinction coefficient, and superior folding property. When ShadowY was paired with mEGFP or a Clover mutant (Clover_T153M/F223R_) and applied to a single-molecule FRET sensor to monitor a light-dependent conformational change of the light-oxygen-voltage domain 2 (LOV2) in HeLa cells, we observed a large FRET signal change with low cell-to-cell variability, allowing for precise measurement of individual cell responses. In addition, an application of ShadowY to a separate-type Ras FRET sensor revealed an EGF-dependent large FRET signal increase. Thus, ShadowY in combination with mEGFP or Clover_T153M/F223R_ is a promising FLIM-FRET acceptor.

## Introduction

Protein conformational changes and protein–protein interactions form the basis of intracellular biochemical signal transduction. Fluorescence lifetime imaging microscopy (FLIM)-based Förster resonance energy transfer (FRET) measurement (FLIM-FRET) is one of the powerful methods for imaging of intracellular protein activities such as protein–protein interactions and conformational changes^1–3^. As a pair of fluorescent proteins for FLIM-FRET, enhanced green fluorescent protein (EGFP) as an energy donor and red fluorescent protein (RFP) as an energy acceptor are frequently used^4, 5^. Because this pair has well-separated emission spectra, this combination prevents spectral contamination due to the bleed-through of RFP fluorescence to the EGFP channel. In contrast to this advantage, because the spectral overlap between the EGFP emission and RFP excitation spectra is relatively small, Förster distance is also relatively short^6^. Furthermore, these fluorescent proteins occupy a wide range of wavelengths (500**–**650 nm), which makes it difficult to use additional fluorescent dyes for multi-color imaging.

FLIM-FRET requires only donor fluorescence (not acceptor fluorescence) for the detection of FRET^5^. By means of this feature, a fluorescent protein with a low quantum yield called resonance energy-accepting chromoprotein (REACh) was developed and applied to an acceptor of FRET^7^. Because this protein has significant absorption properties, it can be used as an acceptor of FRET. When REACh is paired with EGFP, there are three advantages over the EGFP–RFP pair. First, because the spectral overlap of EGFP emission and REACh absorption is larger, the Förster distance is longer (5.6–6.2 nm)^7, 8^ than those of the EGFP–mRFP1/DsRed/mCherry pairs (~4.7–5.3 nm)^9–11^ allowing us to detect the FRET signal in a long range. Second, because REACh has only weak fluorescence, the spectral separation between EGFP and REACh emission is not required, enabling us to utilize the whole wavelength range of EGFP fluorescence (500**–**600 nm) as a signal. Third, because only EGFP fluorescence is present, multicolor imaging using another fluorescent protein such as RFP is possible^7^.

Almost a decade ago, an improved version of REACh called super REACh (sREACh) was designed by introducing several mutations, and the pairing of this protein with A206K-mutated monomeric EGFP (mEGFP) successfully improved the FRET signal because of enhanced maturation efficiency of sREACh in cells^12^. Although mEGFP–sREACh pair yields a superior FRET signal, the spectral contamination of basal sREACh fluorescence owing to residual quantum efficiency (0.07) can produce unexpected artifacts, limiting applications of this pair^8^. To overcome this limitation, a very dark fluorescent protein named ShadowG was developed by directed evolution using sREACh^8^. Although this protein has superior darkness and minimized response variability, the FRET signal is relatively small, compared with that of sREACh^8^.

Here, we aimed to develop a dark yellow fluorescent protein that has high absorption and a low quantum yield as compared with sREACh to increase the FRET signal and prevent an artifact due to residual fluorescence. Saturation mutagenesis at the positions surrounding the chromophore of sREACh led to a new fluorescent protein named ShadowY that has a 7-fold lower quantum yield and a 1.2-fold greater extinction coefficient than those of sREACh. Furthermore, we confirmed that the pairing of ShadowY with mEGFP, Clover^13^, or its mutant (Clover_T153M/F223R_) shows improved FRET signals with reduced cell-to-cell variability. Thus, mEGFP–ShadowY and Clover mutant–ShadowY are good FLIM-FRET pairs.

## Results

**Figure 1:**
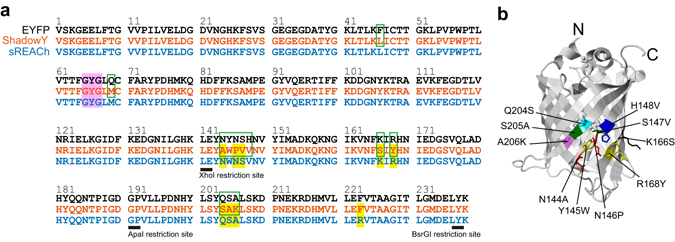
Sequence alignment of EYFP, ShadowY, and sREACh. (**a**) Green rectangles indicate the differences between ShadowY and EYFP. Yellow boxes indicate the differences between ShadowY and sREACh. The chromophore tripeptide is highlighted with the magenta box. (**b**) X-ray crystal structure of EYFP (Protein Data Bank ID: 3V3D). The mutated amino acid positions except F46L and Q69M are indicated.

To create a dark yellow fluorescent protein, we applied saturation mutagenesis to amino acid positions N144, N146, S147, and V148 surrounding the chromophore in the previously reported dark yellow fluorescent protein, sREACh (Fig. 1)^12^. Because position W145 is crucial for reduction of quantum efficiency^7^, we avoided introducing a mutation at this position. We introduced the A206K monomeric mutation, while F223R was reversed (Fig. 1a); A206K increases the dissociation constant more than F223R does^14^. The PCR products with saturated mutations were ligated into a bacterial expression vector, and we thus constructed a genetic library. To screen the library for dark yellow fluorescent proteins that have a high extinction coefficient but a low quantum yield, we first identified vividly colored colonies under day light, confirming that the mutants have high absorption. Subsequently, the colonies were confirmed to have no fluorescence under blue light by a method similar to the one described elsewhere^8, 15^. This screening identified a burnt orange colony under day light but no visible fluorescence under blue light. As a result, sequence analysis identified the following mutations: N144A, N146P, S147V, H148V, A206K, and R223F in sREACh (Fig. 1, Table 1). This mutant named sREACh#1 has a ~2-fold smaller quantum yield, while its extinction coefficient is comparable to that of sREACh (Table 1). Subsequently, with sREACh#1 as a template, saturation mutagenesis at positions Q204/S205/L207 located near the chromophore was carried out. After the screening, spectroscopic and sequence analysis revealed that one of the mutants named sREACh#2 has ~1.14-fold increased extinction coefficient and ~1.5-fold decreased quantum efficiency (Table 1). A similar procedure was also carried out at position K166/R168/H169, and we identified a mutant that shows improved darkness and absorption relative to sREACh#2 (Table 1). We named this mutant *ShadowY* where Y stands for “yellow”, and decided to pursue further analyses of this protein.

**Table 1:** Characteristics of ShadowY.

Protein	EC (M^−1^cm^−1^)	QE	Ex (nm)	Em (nm)	Folding t_1/2_ (sec)	Oxidation t_1/2_ (min)	Fluorescence lifetime (ns)	
							purified	in cells
ShadowG	89,000*	0.005 *	486*	510*	37*	76*	0.16*	ND
sREACh	115,000*	0.07*	517	531*	267	130	0.67	0.54
sREACh (#1)	114,000	0.03	518	530	149	ND	0.37	0.33
sREACh (#2)	130,000	0.02	519	531	75	ND	0.25	0.22
ShadowY	136,000	0.01	519	531	73	140	0.19	0.17

EC: extinction coefficient, QE: quantum efficiency, Ex: excitation maximum, E_m_: emission maximum. *Values obtained from previously published data^8^. sREACh#1 contains the following mutations in sREACh: N144A, N146P, S147V, A206K, and R223F. sREACh#2 contains the following mutations in sREACh#1: Q204S and S205A. ShadowY contains the following mutations in sREACh#2: K166S and R168Y. ND: not determined.

**Figure 2:**
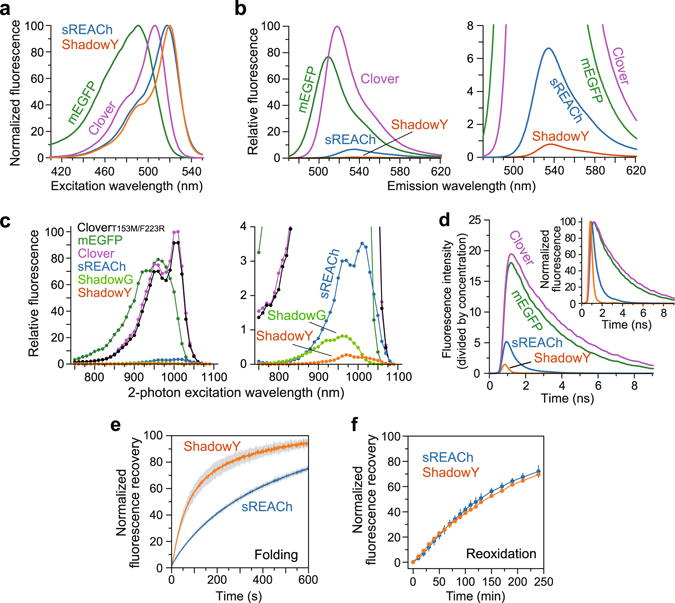
Spectrofluorimetric analysis of purified ShadowY. (**a**) Normalized excitation spectra of mEGFP, Clover, sREACh, and ShadowY. (**b**) The emission spectra of mEGFP, Clover, sREACh, and ShadowY excited at 450 nm. For all the samples, optical density at 450 nm is adjusted to the same value. (right) An enlarged view of left panel. (**c**) Two-photon excitation spectra of 5 µM mEGFP, 5 µM Clover, 5 µM Clover_T153MF223R_, 30 µM sREACh, 100 µM ShadowG, 100 µM ShadowY. The measured fluorescence intensities were divided by the respective protein concentrations, and the peak intensity of Clover was normalized to 100. (right) An enlarged view of left panel. (**d**) Fluorescence lifetime curves of the fluorescent proteins subjected to 2-photon excitation at 920 nm. Measured fluorescence intensity was divided by the respective protein concentration. (inset) Normalized fluorescence lifetime curves. (**e**,**f**) Fluorescence recovery of sREACh and ShadowY from a denatured (**e**) or reduced state (**f**). The respective fluorescent protein was excited at 517 nm with 5 nm bandwidth, and its fluorescence recovery was monitored at 531 nm with 5 nm bandwidth. Three independent experiments were averaged (the data are shown as mean ± SEM).

Spectral analysis of purified ShadowY confirmed that it has an excitation peak at 519 nm and an emission peak at 531 nm (Fig. 2a,b and Table 1), similar to those of sREACh (Table 1). Further analysis revealed that the molar extinction coefficient of ShadowY is 136,000 M^−1^ cm^−1^: a 1.2-fold greater extinction coefficient than that of sREACh (Table 1). Quantum efficiency of ShadowY is 0.01, which is 7-fold smaller than that of sREACh (QE, 0.07; Table 1). Consistent with these results, two-photon excitation spectrum of ShadowY exhibited the low fluorescence compared with those of mEGFP and Clover (Fig. 2c), and the fluorescence lifetime of ShadowY (0.19 ns) is much shorter than that of sREACh (0.67 ns; Table 1, Fig. 2d).

We next characterized the folding and maturation kinetics of ShadowY by the urea-denaturation method as described previously^16^. The fluorescence of denatured ShadowY recovered in 73 sec: faster than recovery of sREACh (267 sec; Table 1, Fig. 2e), suggesting that ShadowY has superior folding properties. Next, chromophores of the urea-denatured ShadowY were reduced with dithionite, and reoxidation time and recovery were monitored after dilution in urea-free buffer. Reoxidation time of ShadowY (140 min) is comparable to that of sREACh (130 min; Fig. 2f, Table 1).

**Figure 3:**
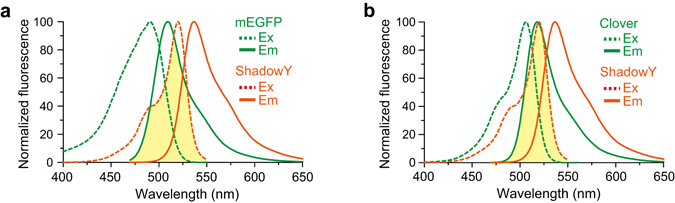
The spectral overlap of fluorescent proteins. (**a**,**b**) The spectral overlap (yellow region) between ShadowY’s excitation spectrum and mEGFP’s (**a**) or Clover’s (**b**) emission spectra.

**Figure 4:**
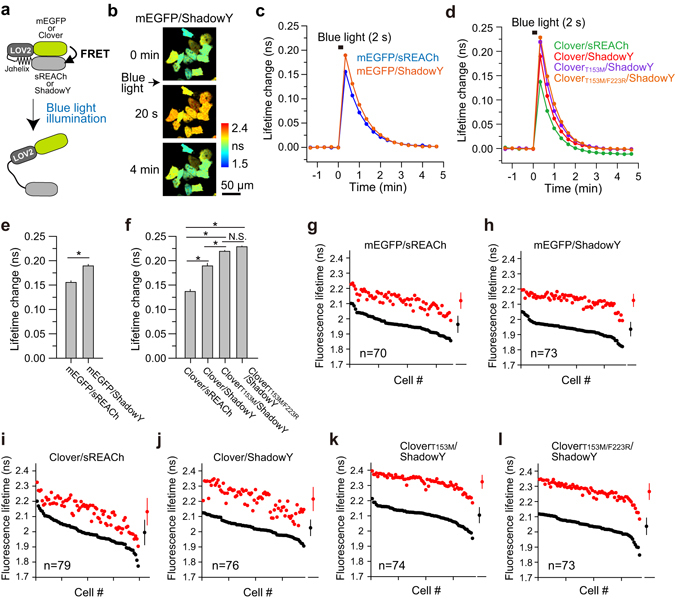
Performance of ShadowY in LOV2 FRET sensors in HeLa cells. (**a**) A schematic of a conformational change of the light-sensitive LOV2 FRET sensors. (**b**) Representative fluorescence lifetime images of mEGFP-LOV2-ShadowY after blue light illumination for 2 seconds at 35 mW/cm﻿^2﻿^. The scale bar is 50 µm. (**c**) An averaged time course of fluorescence lifetime changes in response to blue light illumination. The number of cells analyzed is 70 for mEGFP–sREACh and 73 for mEGFP–ShadowY. The data are presented as mean ± SEM. (**d**) An averaged time course of fluorescence lifetime changes in response to blue light illumination. The number of cells analyzed is 79 for Clover–sREACh, 76 for Clover–ShadowY, 74 for Clover_T153M_–ShadowY, and 73 for Clover_T153MF223R_–ShadowY. The data are presented as mean ± SEM. (**e**,**f**) The fluorescence lifetime changes at 20 sec after blue light illumination were quantified using the data presented in (**c**,**d**), respectively. The data are presented as mean ± SEM. Asterisks denote statistical significance (p < 0.05, analysis of variance [ANOVA] followed by Scheffé’s *post hoc* test). N. S. = not statistically significant. (**g–l**) The lifetime changes in individual HeLa cells before and after light illumination (the same dataset as in panels c and d). The basal fluorescence lifetime (averaged over −1.3 to 0 min) of individual cells is plotted in the descending order (black) along with the corresponding fluorescence lifetime values (at 20 sec) after blue-light illumination (red). The data are also presented as mean ± SD on the right. The number of samples (n) is indicated in respective panels.

Next, we tested the performance of ShadowY as an energy acceptor for 2-photon FLIM-FRET via comparison with sREACh in HeLa cells. We used 2-photon excitation for imaging because of the reduced phototoxicity compared with 1-photon excitation^17^. We chose mEGFP or Clover as an energy donor, because the emission spectra of these proteins significantly overlap with the excitation spectrum of ShadowY (Fig. 3a,b). To quantify the performance of mEGFP–ShadowY and Clover**–**ShadowY pairs in comparison with mEGFP–sREACh and Clover–sREACh pairs, we fused these fluorescent proteins to the N and C termini of a light-sensitive LOV2-Jα helix domain from Phototropin 1^18, 19^, respectively, creating mEGFP-LOV2-ShadowY, mEGFP-LOV2-sREACh, Clover-LOV2-ShadowY, and Clover-LOV2-sREACh as LOV2 FRET sensors (Fig. 4a), and monitored the blue-light-dependent structural change in HeLa cells by means of 2-photon FLIM-FRET (Fig. 4a,b). HeLa cells expressing the LOV2 FRET sensor were illuminated with blue light at 35 mW/cm^2^ for 2 sec (Fig. 4b–d). Right after illumination, the fluorescence lifetime of mEGFP in LOV2 FRET sensors increased, i.e., FRET decreased, and returned in ~60 sec, consistent with another study^19^. The quantitative analysis indicated a significant increase in the fluorescence lifetime change of mEGFP-LOV2-ShadowY relative to mEGFP-LOV2-sREACh (Fig. 4e). Furthermore, we compared the cell-to-cell variability of FRET signals (Fig. 4g,h), and found that the variability of mEGFP-LOV2-ShadowY in both the basal state and after light illumination (before, 1.94 ± 0.05 ns; after, 2.13 ± 0.04 ns) is smaller than that of mEGFP-LOV2-sREACh (before, 1.96 ± 0.06 ns; after, 2.12 ± 0.05 ns). Taken together, these results suggest that ShadowY is superior FLIM-FRET acceptor.

Next, we compared Clover-LOV2-ShadowY and Clover-LOV2-sREACh and found that Clover-LOV2-ShadowY shows larger lifetime change and smaller cell-to-cell variability (before, 2.03 ± 0.05 ns; after, 2.21 ± 0.08 ns) than Clover-LOV2-sREACh does (before, 1.99 ± 0.08 ns; after, 2.13 ± 0.09 ns; Fig. 4d,f,i,j). Nevertheless, when Clover-LOV2-ShadowY was compared with mEGFP-LOV2-ShadowY, the cell-to-cell variability of cells expressing Clover-LOV2-ShadowY, especially after light illumination (after, 2.21 ± 0.08 ns; Fig. 4j), was larger than that of mEGFP-LOV2-ShadowY (after, 2.13 ± 0.04 ns; Fig. 4h). Therefore, we attempted to improve cell-to-cell variability by introducing mutations into Clover. We speculated that the difference in cell-to-cell variability originates from the difference in amino acid sequence between mEGFP and Clover, especially the amino acids whose side chains are outward-directed. First, using EYFP crystal structure (Fig. 1b), we confirmed that the side chains of amino acids located at R30, N39, S99, T105, T153, and A206 in Clover are outward-directed and are different from those of mEGFP. Next, each of the above amino acid residues was reverted to the residue corresponding to mEGFP, i.e., R30S, N39Y, S99F, T105N, T153M, and A206K, respectively. Among these mutations, T153M improved the lifetime change and reduced the cell-to-cell variability (before, 2.10 ± 0.05 ns; after, 2.32 ± 0.05 ns; Fig. 4k), compared with Clover/ShadowY pair. Furthermore, to increase monomericity, we introduced the F223R monomeric mutation into Clover_T153M_^14^. Resultant construct Clover_T153M/F223R_-LOV2-ShadowY showed performance that was similar to that of Clover_T153M_-LOV2-ShadowY (before, 2.04 ± 0.06 ns; after, 2.27 ± 0.05 ns; Fig. 4l). Spectral analysis of purified Clover mutants confirmed that Clover_T153M/F223R_ has a comparable extinction coefficient and quantum efficiency with those of Clover and Clover_T153M_ (Table 2). Moreover, Förster distance between Clover_T153M/F223R_ and ShadowY was 6.4 nm, comparable with that of mEGFP–ShadowY and Clover–ShadowY pairs (Table 2).

**Table 2:** Characteristics of Clover mutants.

Protein	EC (M^−1^cm^−1^)	QE	Ex (nm)	Em (nm)	Fluorescence lifetime (ns)		Förster distance with	
					purified	in cells	ShadowY (nm)	sREACh (nm)
mEGFP	58000*	0.73*	488*	507*	2.73	2.60	6.2	6.2*
Clover	111000^†^	0.76^†^	505^†^	515^†^	3.29	3.11	6.3	6.3
Clover_T153M_	112000	0.80	505	515	3.34	3.11	6.4	6.4
Clover_T153M/F223R_	122000	0.81	505	515	3.31	3.08	6.4	6.4

EC: extinction coefficient, QE: quantum efficiency, Ex: excitation maximum, E_m_: emission maximum. ^*,†^Values obtained from previously published data^8, 13^, respectively. For the calculation of Förster distance, random interfluorophore orientation were assumed ^30^. ND: not determined.

**Figure 5:**
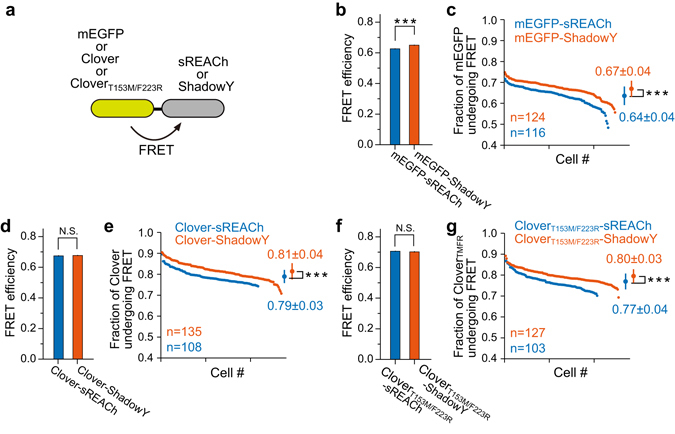
FRET efficiency and maturity of ShadowY in tandem fluorescent proteins. (**a**) A schematic drawing of the tandem fluorescent protein used to evaluate the FRET efficiency and fraction of the donor fluorescent protein undergoing FRET (chromophore maturation efficiency) for ShadowY. (**b**,**d**,**f**) Comparison of FRET efficiency of the tandem fluorescent proteins. The fluorescence lifetime decay curve averaged over the whole image was used for the analysis (See *Materials and Methods*). The number of images used for the analysis is 10–13. Each image contains 8–15 cells, and the data are presented as mean ± SEM. Asterisks denote statistical significance (*t* test, *P < 0.05, **P < 0.01, ***P < 0.001, N.S. = not significant). (**c**,**e**,**g**) A comparison of the fraction of donor fluorescent protein undergoing FRET analyzed in individual cells and data ﻿was﻿ plotted in the descending order. The FRET fraction is directly related to the maturation efficiency of an acceptor, i.e., sREACh or ShadowY. Means ± SD are also plotted on the right (*t* test, *P < 0.05, **P < 0.01, ***P < 0.001, N.S. = not significant). The number of samples (n) and mean ± SD are also indicated in figures.

To further characterize ShadowY, we measured FRET efficiency and maturation efficiency using tandem fluorescent proteins in HeLa cells as described previously^8^. We expressed tandem constructs (Fig. 5a), and the fluorescence lifetime of mEGFP, Clover, or Clover_T153M/F223R_ was measured by 2-photon FLIM-FRET (Fig. 5b–g). Because the fluorescence lifetime decay curves are convolution of both the FRET efficiency and maturity of an acceptor^5^, we measured these parameters separately, as described earlier^2, 12^. Although FRET efficiencies of all the compared pairs showed comparable values (Fig. 5b,d,f), the maturity of ShadowY was found to be slightly better than that of sREACh in all pairs (Fig. 5c,e,g).

**Figure 6:**
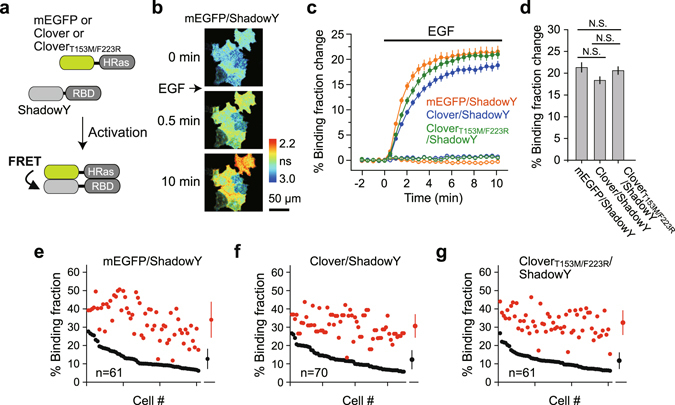
The performance of ShadowY in H-Ras FRET sensors in HeLa cells. (**a**) A schematic of the H-Ras FRET sensor activation. (**b**) Representative fluorescence lifetime images of mEGFP-H-Ras paired with ShadowY fused to the Ras-binding domain (RBD) in HeLa cells after stimulation with 50 nM EGF. The scale bar is 50 µm. (**c**) An averaged time course of binding fraction changes in response to EGF stimulation (filled circles) or with empty buffer (open circles; control). Cells showing a 5–30% basal binding fraction (before stimulation) were chosen for the analysis. The number of cells analyzed is 61 for mEGFP–ShadowY, 70 for Clover–ShadowY, and 61 for Clover_T153M/F223R_–ShadowY. In the control, the number of cells analyzed is 45 for mEGFP–ShadowY, 18 for Clover–ShadowY, and 31 for Clover_T153M/F223R_–ShadowY. The data are presented as mean ± SEM. (**d**) Binding fraction changes (averaged over 8 to 10 min) after EGF stimulation. The data are presented as mean ± SEM; N. S. = not statistically significant (p > 0.05, analysis of variance [ANOVA] followed by Scheffé’s *post hoc* test). (**e**–**g**) Activation of H-Ras in individual HeLa cells after stimulation with EGF (the same dataset as in panel c). The basal binding fraction (averaged over −2 to 0 min) of individual cells is plotted in the descending order (black) along with the corresponding binding fraction (averaged over 8 to 10 min) after EGF stimulation (red). The data are also presented as mean ± SD on the right.

Next, mEGFP–ShadowY, Clover–ShadowY, and Clover_T153M/F223R_–ShadowY pairs were applied to a separate-type H-Ras FRET sensor^8, 20^, and their FRET signals were compared (Fig. 6). We did not compare with sREACh because it has a bleed-thorough effect (Fig. S1). As a FRET donor, H-Ras was fused to mEGFP, Clover, or Clover_T153M/F223R_, and as an acceptor, the Ras-binding domain of Raf1 was fused to ShadowY (Fig. 6a). The donor and acceptor were fused via the P2A sequence to ensure equal expression of these molecules^21^ and to minimize the response variability due to the imbalanced expression of the donor and acceptor. We transfected HeLa cells with these FRET sensors and compared their response signals as a binding fraction change (Fig. 6b–g). After stimulation with epidermal growth factor (EGF), H-Ras was rapidly activated (within a few minutes; Fig. 6b,c). When the FRET response signals of Ras sensors were compared, all the three FRET sensors showed a similar signal change (Fig. 6d–g), with the values of 21.34 ± 1.05 (mEGFP–ShadowY), 18.39 ± 0.77 (Clover–ShadowY), 20.62 ± 0.86 (Clover_T153M/F223R_–ShadowY), respectively (Fig. 6d).

## Discussion

Here, we successfully developed a new dark yellow fluorescent protein, ShadowY, as a FLIM-FRET acceptor for pairing with mEGFP or the Clover mutant. ShadowY has superior properties in terms of absorption and folding kinetics relative to sREACh (Fig. 2, Table 1). These factors most likely contribute to the increased FRET signals and the reduced cell-to-cell variability, compared with those of sREACh (Figs 4 and 5). Furthermore, although sREACh is difficult to apply to a separate-type FRET sensor because of bleed-through fluorescence contamination^8^, ShadowY does not have this problem because of the superior darkness relative to sREACh (Fig. S1). An application of ShadowY to an LOV2 and H-Ras FRET sensors yielded a large FRET change (Figs 4c,d and 6c), which is larger than that of the previously reported mCherry or ShadowG version of sensors^8^.

In the past decade, several types of dark fluorescent proteins have been identified and applied to FRET imaging, photoacoustic imaging, and structural analysis^7, 8, 12, 22–25^. We believe that ShadowY will be an additional useful tool for these studies, especially for FLIM-FRET.

## Materials and Methods

### Saturation mutagenesis

The *sREACh* gene in a customized pRSET vector (Invitrogen) served as an initial template for construction of genetic libraries. First, a *XhoI* restriction site was silently introduced at the positions corresponding to amino acid residues L141 and E142 in *sREACh* (Fig. 1a). Saturated mutagenesis was performed by PCR amplification of *sREACh* (the fragment corresponding to amino acid positions 141–238) with degenerate primers. These primers are as follows: For sREACh#1, FW 5′-gagactcgagtacNNBtggNNBNNBNNBNNBgtctatatcatggccga-3′, RV 5′-gagaggatcccttgtacagctcgtccat-3′, and *Xho*I and *BsrG*I are used for subcloning into the custom pRSET vector; for sREACh#2, FW 5′-gagagggcccgtgctgctgcccgacaaccactacctgagctacNNBNNBaagNNBagcaaagaccccaacg-3′, RV 5′-gagaggatcccttgtacagctcgtccat-3′, and *Apa*I and *BsrG*I sites were used for subcloning; for ShadowY, FW 5′-gagactcgagtacgcttggcccgtggtgaatgtctatatcatggccgacaagcagaagaacggcatcaaggtgaacttcNNKatcNNKNNKaacatcgaggacggca-3′, RV 5′-gagaggatcccttgtacagctcgtccat-3′, and *Xho*I and *BsrG*I sites were used for subcloning. The plasmid was then introduced into electrocompetent cells, and the cells were grown for 18**–**20 h at 34 °C on LB agar plates supplemented with antibiotics.

### Plasmid construction

In all DNA construction procedures described below, a modified pEGFP-C1 plasmid (Clontech) served as a backbone vector. For construction of the EGFP or Clover with the CAAX motif of K-Ras (corresponding to amino acid residues 173–188), EGFP or Clover fused to CAAX via a linker encoding the peptide SGLRSRAQASNSAV was inserted into the vector by replacing EGFP. To create cytosolic mCherry, sREACh, or ShadowY as shown in Fig. S1, the EGFP in the vector was replaced by the respective genes. To create the tandem fluorescent protein constructs (shown in Fig. 5), the respective combination of fluorescent proteins was fused with a linker encoding the peptide SGLRSG in the vector.

For construction of the LOV2 FRET sensor, a donor fluorescent protein was fused to the N terminus of the LOV2 domain (DNA sequence corresponding to amino acid residues 404–546 in Phototropin 1) via a linker encoding the peptide ASM. The acceptor fluorescent protein was fused to the C terminus of the LOV2 domain via the linker peptide KLGNS.

For construction of the H-Ras FRET sensors, we fused an acceptor fluorescent protein to the C terminus of the Ras-binding domain of Raf1 (amino acid residues 50–131 with two mutations: K65E and K108A)^20^ via the linker peptide GSG. Subsequently, H-Ras fused to a donor fluorescent protein via the linker peptide SGLRSRG was fused to the C terminus of the acceptor protein via the P2A sequence^21^ so that the Ras-binding domain and H-Ras parts were translated into different polypeptides within the cell.

### Fluorescent properties of the fluorescent proteins

His-tagged fluorescent proteins were overexpressed in *Escherichia coli* DH5α cells using a modified pRSET vector (Invitrogen) and purified on a Ni^+^-nitrilotriacetate column (HiTrap, GE Healthcare). Excitation and emission spectra of the fluorescent proteins diluted in PBS were recorded on a spectrofluorometer (RF-6000; Shimadzu). Matured-protein concentrations were calculated from the extinction coefficient of the chromophore after denaturation in 0.1 N NaOH (40,000 M^−1^ cm^−1^ at 446 nm)^26^. The extinction coefficients of fluorescent proteins were determined by dividing the peak optical density by the molar concentration of matured proteins. Quantum efficiency of the proteins was determined by a comparison with that of Clover (0.76) as described elsewhere^13^.

Two-photon excitation spectra were measured under the two-photon fluorescence microscope (FVMPE-RS; Olympus). An Insight Ti:Sapphire laser (Spectra-Physics) with the power of 3.4–4.5 mW at the respective wavelength under the objective lens was used to excite the purified fluorescent proteins. Raw fluorescence intensity values were corrected by dividing them by squared laser power used for each wavelength.

### Refolding and reoxidation

To measure the refolding time of ShadowY after denaturation, the proteins were dissolved in denaturation buffer (8 M urea, 1 mM dithiothreitol) and heated at 95 °C for 5 min as described previously^16^. The refolding was initiated by diluting the denatured protein with a 100-fold volume of renaturation buffer (5 mM KCl, 2 mM MgCl_2_, 50 mM Tris-HCl pH 7.5, 1 mM dithiothreitol) at room temperature. For the reoxidation experiment, 5 mM dithionite was added into the denaturation buffer to reduce the chromophore. The respective fluorescent protein was excited at 517 nm with 5 nm bandwidth, and its fluorescence recovery was monitored at 531 nm with 5 nm bandwidth in a spectrofluorometer (RF-6000; Shimadzu).

### Cell culture and transfection

HeLa cells were cultured in Dulbecco’s modified Eagle’s medium (DMEM; supplemented with 10% of fetal bovine serum) at 37 °C and 5% of CO_2_. The cells were transfected with the plasmids by means of Lipofectamine 3000 (Invitrogen), followed by incubation for 16–20 h. FLIM-FRET imaging was conducted in a solution containing 4-(2-hydroxyethyl)-1-piperazineethanesulfonic acid (HEPES; 30 mM, pH 7.3)-buffered artificial cerebrospinal fluid (130 mM NaCl, 2.5 mM KCl, 1 mM CaCl_2_, 1 mM MgCl_2_, 1.25 mM NaH_2_PO_4_, 25 mM glucose) at room temperature.

### Two-photon fluorescence lifetime imaging

Details of the 2-photon FLIM-FRET imaging were described elsewhere^5, 8^. Briefly, mEGFP or Clover in the FRET sensor was excited with a Ti-sapphire laser (Mai Tai; Spectra-Physics) tuned to 920 nm. The scanning mirror was controlled with the ScanImage software^27^. The green fluorescence photon signals were collected by an objective lens (60 × , 0.9 NA; Olympus) and a photomultiplier tube (H7422-40p; Hamamatsu) placed after a dichroic mirror (565DCLP; Chroma) and emission filter (FF01-510/84 or FF03-510/20 in Fig. S1; Semrock). Measurement of fluorescence lifetime was conducted using a time-correlated single-photon counting board (SPC-150; Becker & Hickl) controlled with custom software^5^. For construction of a fluorescence lifetime image, the mean fluorescence lifetime in each pixel was translated into a color-coded image^2, 28^. Analysis of the lifetime change and binding-fraction change was conducted as described elsewhere^2, 29, 30^. In Fig. 4, blue LED (244-87-470-50E-40; CoolLED) with a band pass filter (FF01-469/35-25; Chroma) was used for illumination to induce the structural change of LOV2 FRET sensors.

### Analysis of the fluorescence lifetime image

To generate fluorescence lifetime images, we acquired the mean fluorescence lifetime in each pixel by calculating the mean photon arrival time <*t*>as

$$\langle t\rangle =\int tF(t)dt\div\int F(t)dt-{t}_{0}$$

where *t*_*o*_ is obtained by fitting the whole image with single exponential or double exponential functions convolved with an instrument response function as described previously^2, 30^. After that, the mean fluorescence lifetime in each pixel was converted to the corresponding color. FRET efficiency and the fraction of the donor fluorescent protein undergoing FRET were calculated as in other studies^2, 5, 8, 30^.

## Electronic supplementary material

Supplementary information
