# Supplementary material for: ShadowY: a dark yellow fluorescent protein for FLIM-based FRET measurement
Source: Sci Rep. 2017 Jul 28;7:6791. doi: 10.1038/s41598-017-07002-4 (PMC5533704; doi:10.1038/s41598-017-07002-4)
Supplement: Supplementary file 1 — Supplementary information [file 41598_2017_7002_MOESM1_ESM.pdf]

Supplementary information

ShadowY: a dark yellow fluorescent protein for FLIM-based FRET measurement

Hideji Murakoshi[1,2,3,]* and Akihiro C. E. Shibata[1]

[1] Supportive Center for Brain Research, National Institute for Physiological Sciences, Okazaki, Aichi 444-8585, Japan, [2] Department of Physiological Sciences, SOKENDAI (The Graduate University for Advanced Studies), Okazaki, Aichi 444-8585, Japan, [3] Precursory Research for Embryonic Science and Technology, Japan Science and Technology Agency (JST), Kawaguchi, Saitama 332-0012, Japan

*Correspondence author: Hideji Murakoshi; e-mail: murakosh@nips.ac.jp; Tel.: +81 564-55-7857; Fax: +81 564-55-7858

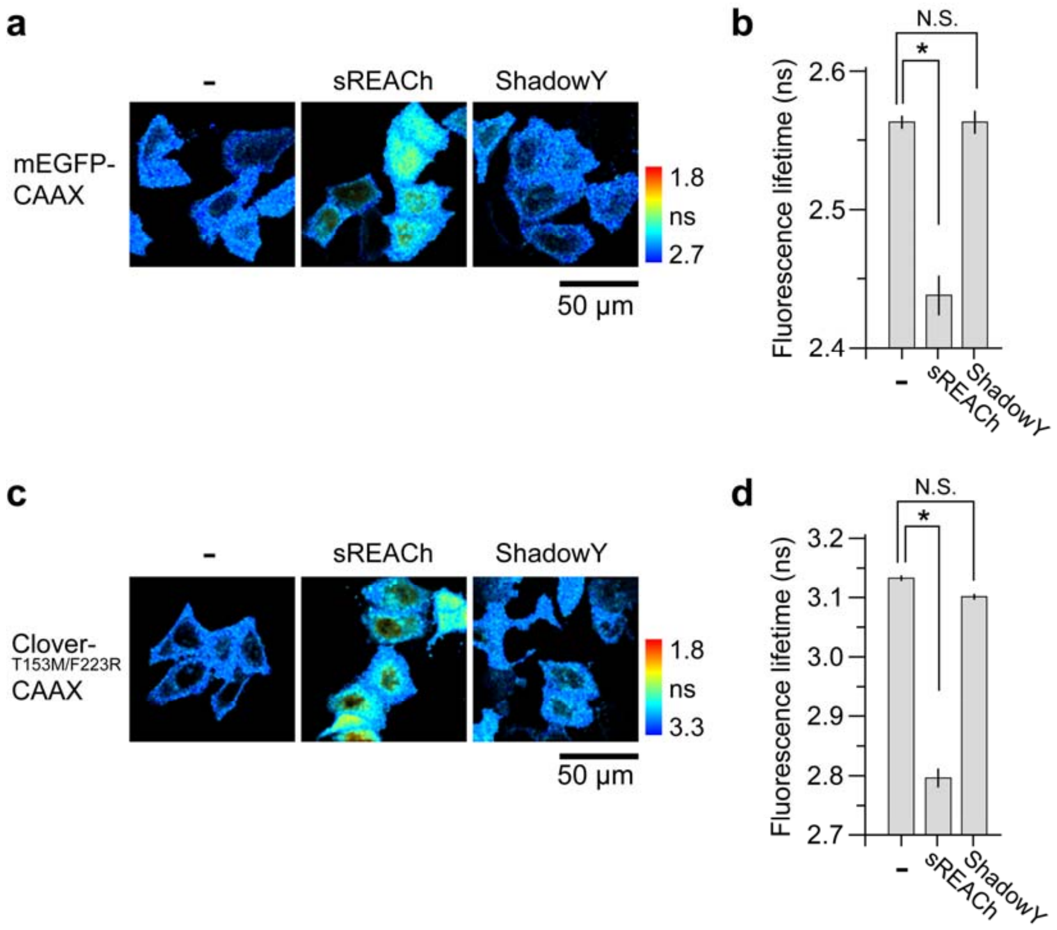

Figure S1. Measurement of fluorescence contamination of sREACh and ShadowY.

(a, c) Fluorescence lifetime images of HeLa cells expressing CAAX (a motif of K-Ras) fused to mEGFP (a) or Clover$_{T153MF223R}$ (c), which is localized to the plasma membrane. The cells also express cytosolic sREACh or ShadowY, assuming that these proteins do not interact with either mEGFP or Clover$_{T153M/F223R}$. Two-photon excitation at 920 nm was used for the measurement. Redder color indicates fluorescence contamination with short lifetime (sREACh: 0.67 ns, ShadowY: 0.19 ns; Table 1). The scale bar is 50 µm.

(b, d) Quantitative comparison of the fluorescence lifetime of cells expressing mEGFP-CAAX (b) or Clover$_{T153M/F223R}$-CAAX (d) along with the empty vector, sREACh, or ShadowY. The fluorescence lifetime decay curve averaged over the whole image was analyzed. The number of images is 10 for all conditions. Each image contains 4–10 cells. The data are presented as mean ± SEM. Asterisks denote statistical significance ($p < 0.05$, analysis of variance [ANOVA] followed by Scheffé's *post hoc* test; N.S. = not significant).
